# Changes in immune system and intestinal bacteria of cows during the transition period

**DOI:** 10.1016/j.vas.2021.100222

**Published:** 2021-12-02

**Authors:** S. Chida, M. Sakamoto, T. Takino, S. Kawamoto, K. Hagiwara

**Affiliations:** a School of veterinary Medicene, Rakuno Gakuen University, 582 Bunkyodai Ebetsu, Hokkaido, 069-8501 Japan; b Scientific Feed Laboratory co., ltd., R & D center, Sakura city, Chiba, 285-0043 Japan

**Keywords:** CD, cluster of differentiation, IL, Interleukin, LPS, Lipopolysaccharide, TMR, Total-Mixed-Ration, TDN, Total-Digestible-Nutrients, EDTA, ethylenediaminetetraacetic acid, PBMC, peripheral blood mononuclear cell, TNF, Tumor Necrosis Factor, TGF, Transforming Growth Factor, GAPDH, Glyceraldehyde 3-phosphate dehydrogenase, Dairy cows, High-energy feed, Probiotics, *Lactobacillus plantarum*, Anti-inflammatories

## Abstract

High-yield dairy cows need high energy feed during periods of increased milk production. The transitional feeding to high energy feed increases the risk of developing a variety of metabolic disorders. Here, five Holstein cows were fed a four-stage feeding protocol (3 weeks for each stage) ranging from 54.9 to 73.7% total digestive nutrients (TDN). The purpose of the study was to investigate the effect of lactic acid bacteria on high-energy-fed cows associated with transitional feeding, and to evaluate the effects of probiotics on intestinal bacterial changes and inflammatory responses. Three feed transition periods were established for five cows, and *Lactobacillus plantarum* RGU-LP1 (LP1) was fed as a probiotic during the high-energy feeding period. The number of lymphocyte subsets such as CD3-, CD4-, and CD8 positive cells decreased in response to the high energy feed. Lipopolysaccharide (LPS)-induced cytokine (*IL-1β* and *IL-2*) gene expression in peripheral blood mononuclear cells (PBMCs) was shown to increase in those animals receiving the high energy feed. However, supplementation with LP1 resulted in an increase in the number of lymphocyte subsets and the expression of *IL-1β* and *IL-2* were returned to the level at low energy diet. These results suggest that high energy diets induce inflammatory cytokine responses following LPS stimulation, and that the addition of LP1 mitigates these results by regulating the LPS-induced inflammatory reaction. Therefore, the functional lactic acid bacteria LP1 is expected to regulate inflammation resulting from high energy feeding, and this probiotic could be applied to support inflammatory regulation in high-yield dairy cows.

## Highlights

• Transitional high-energy diets reduce peripheral blood lymphocytes in dairy cows.
• High-energy diets upregulate IL-1*β* and IL-2 and downregulate IL-10 expression.
• Functional lactobacillus plantarum LP1 restores normal levels of lymphocytes subset.
• Lactobacillus plantarum LP1-added diets reduce inflammatory cytokine expression.
• LP1 mitigates immune response imbalances caused by transitional high energy diets.

## Introduction

Dairy cattle feeds need to account for the increasing energy requirements of increased lactation and increased milk production in the early lactation period. Lactation may result in a sizable energy gap that must be filled to ensure further production, this means that most dairy cows will receive some form of high-concentrate grain during lactation. If the feed composition does not change, the energy requirement is not met. Animals in the early stages of lactation should be fed high levels of grain and placed in a feeding stage that allows for the positive regulation of lactation while reducing the negative energy balance associated with milk production (Harder, Stamer, Junge & Thaller, 2019; Krizsan, Sairanen, Höjer & Huhtanen, 2014). This is particularly true in animals that demonstrate a high level of lactation (Weiss, 2017). However, high energy diets reduce the pH in the rumen forcing a change in the rumen microbiome which is exacerbated by reduced hay consumption and saliva production (Mackie & Gilchrist, 1979). In addition, subacute rumen acidosis may result in the release of various exotoxins such as LPS when the ruminal bacteria die (Ringseis, Gessner & Eder, 2015). This upregulates liver stress and may induce several serious metabolic diseases including fatty liver disease and ketosis (Andersson, 1988). There is also an overlap between gestation and early lactation which means that these animals must also deal with their gestation stress, placing these animals at high risk for developing several perinatal diseases such as ruminal tympany and abomasal displacement (Welch et al., 2020). This means that nutritional care and controlled feeding is critical to the overall health of dairy cows.

The composition of the intestinal flora is closely related to overall cattle health and is affected by various environmental factors including both feed composition and antibiotic treatment (Mitsuoka, 2014). The intestinal microbiota is also linked to the host immune system and closely associated with immune maturity and allergy control (Hua, Goedert, Pu, Yu & Shi, 2016). On the other hand, IgA production by the regulatory T cells controls the intestinal microbiome. Lactobacillus is the main component in the intestinal microbiome with these microbes regulating the growth of other potentially pathogenic bacteria preventing their negative effect on the host and upregulating the host immune response. Probiotics are defined as any viable microorganisms that exerting some beneficial effect on the host by their improving of the intestinal bacterial balance (Fuller, 1989). Probiotics can contribute to improved host health by helping to maintain a healthy equilibrium between the host and the intestinal microbiome. Probiotic administration, aimed at improving growth potential and immunity, has gained considerable popularity in cow management. In a study of newborn calves with *Lactobacillus plantarum* GBLP-1 (LP), it has been reported that probiotic-administered calves improve their growth potential by minimizing problems such as infectious diseases (Casper, Hultquist & Acharya, 2021). In other reports, feedlot cattle receiving a synbiotic supplement had improved response to BRD treatment (Colombo et al., 2021). Feeding milk replacer-based probiotics to calves provides significant clinical benefits to the prevention and treatment of calf diarrhea (Kayasaki et al., 2021).

Here we conducted a prospectively collected observational follow-up study from five cows under a four-stage transitional feeding (low to high energy diet) protocol. We also examined the effect of *Lactobacillus plantarum* RGU-LP1(LP1) probiotic on the immune response in these same cows under the high energy feeding.

## Materials and methods

### Probiotic bacteria strain

Here we used *Lactobacillus plantarum* RGU-LP1 (LP1, Patent# 35,610,472) as our probiotic strain. *L. plantarum* RGU (Lp1), a functional lactic acid bacterium strain isolated from naturally fermented milk, without any genetic modification. It is characterized by gastric acid resistance, bile acid resistance, low temperature growth, and various sugar assimilating bacteria, and has the effect of enhancing the metabolic activity and immune function of the administered animal.

They were cultured in 10% skim milk (Megmilk Snow Bland Co., Ltd. Hokkaido Japan) inoculated with a single colony and cultured at 37 °C for 72 h. These solutions were then used to produce a bacterial solution at known concentration (10^10^ CFU/mL) in 10% skim milk. These bacterial solutions were stored at 4 °C and used within 3 days.

### Animals and experimental design

The experimental cows are healthy Holstein-Friesian dairy cows aged 3–6 years (dry milk period 2–12 months). These cows are managed on experimental farms at Rakuno Gakuen University as fistula-equipped dairy cows for rumen and metabolic studies. The rumen fluid is measured directly from these bovine fistulas. No other studies were conducted during the study period and for 6 months before and after. This study was approved by the Ethics Committee from the Rakuno Gakuen University in Japan (VH16C10).

**Fig. 1:**
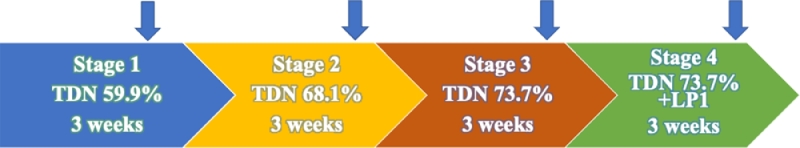
Experimental design of transitional feeding protocol. Examined five cows are fed with each stage feed composition for 3 weeks (show TDN% in the frame). In stage 4, *L plantarum* (Lp1) was administered to all 5 cows under the same feed as stage 3. The arrow indicates the sampling point.

Changes in biometric information using the same cow can be clearly confirmed before and after administration of probiotics. Based on the above intention, in this study, 5 cows were fed a feed from low to high energy diet. In the beginning, the five cows received only hay (15 kg / head / day) for 2 weeks for habituation. This study is an observational follow-up study prospectively collected from five cows under a four-stage transitional feeding protocol (low to high energy diet). We also observed the immune effects of LP1 during the high energy feeding period in the same cows at stage 4 (Fig. 1). The detailed energy composition of the feed is as follows, and the component details are shown in Table 1. The five cows received reduced amounts of hay in their Total-Mixed-Ration (TMR) every three weeks and were fed a high-energy diet in Stage 3. In stage 4, the cows were given LP1 (10^9^ CFU/head/day) and the same TMR as in stage 3. Total-Digestible-Nutrients (TDN) at stages 1 to 3 were 54.9%, 68.2%, and 73.7%, respectively (Fig. 1). The cows were fed each stage of the diet twice a day, morning and evening. Water was provided *ad libitum*. We made daily clinical observations and each cow was tested for a complete blood count using the pocH-100iV Diff (Sysmex Co. Ltd., Hyogo, Japan) system before proceeding to the next feeding phase.

**Table 1:** Diet composition at different the stages.

	Timothy Hay (%)	Concentrate (%)			
		Alfalfa	Steam-flaked corn	DDGS	calcium carbonate for feed
Stage 1 (54.9%)	100	0	0	0	0
Stage 2 (68.1%)	53.48	24.06	21.39	0	1.07
Stage 3 (73.7%)	35.12	30.09	22.73	10.33	0.83

*DDGS: Dried Distiller's Grains with Solubles (%): Total digestible nutrients (TDN).

### Sampling

Blood samples were collected from the jugular vein using a blood collection tube supplemented with ethylenediaminetetraacetic acid (EDTA) for the purpose of immunological blood tests. The peripheral blood mononuclear cell (PBMC) fraction from the blood was then separated by density gradient method using the Ficoll-Conray (density, 1.086). Then the PBMC was washed with RPMI 1640 media for 3 times and used for the immunological examination.

Fecal samples were collected from the rectum and immediately prepared with sterile PBS to prepare a stool diluent and cultured with intestinal bacterial selection. All samples were collected on day 20 of each feeding stage, with rectal feces and blood collected prior to morning feeding. The rumen fluid was collected 3 times a day before feeding in the morning and 5 h and 10 h after feeding. For the rumen fluid, a hollow pipe with transverse holes was inserted through the fistula and 500 ml of the contents were removed from the lower liquid portion by catheter aspiration. Following collection, immediately mesh filtration was performed and pH was measured with a pH meter (LAQ UA twin, Horiba co Ltd., Kyoto, Japan). The average value of three measurements was calculated.

### Intestinal microbiota analysis

Feces were subjected to serial 10-fold dilutions in phosphate-buffered saline (PBS). Diluted samples were cultured on modified *Lactobacillus* selection (LBS; Nissui Pharmaceutical, Tokyo, Japan), *Bifidobacterium* selection (BS; Nissui Pharmaceutical, Tokyo, Japan), and deoxycholate hydrogen sulfide lactose (DHL) agar (Nissui Pharmaceutical, Tokyo, Japan). Coliform colonies were counted after the samples were cultured under aerobic conditions for 24 h using DHL agar. *Lactobacillus sp*. and *Bifidobacterium sp*. colonies were counted after the samples were cultured for 48 h in an anaerobic environment on LBS or BS agar, respectively. The results were expressed as the number of colony-forming units (CFUs) per gram of fecal matter.

### Flow cytometry analysis

Lymphocyte subsets in the peripheral blood samples were evaluated using flow cytometry. PBMCs from each stage were incubated with mouse anti-bovine CD3 (MM1A), CD4 (CACT138A), CD8 (CACT80C) and γδT (WC1; IL-A29)- monoclonal antibodies (Veterinary Medical & Research Development, WA USA) for 45 min at room temperature. The cells were then washed with PBS twice, incubated with rabbit anti-mouse fluorescein isothiocyanate (FITC) antibody (ROCKLAND Inc., PA, USA) for 45 min at 4 °C, then washed with PBS another two times, treated with 0.5% formalin-PBS, and used for flow cytometry analysis (EPICS XL, Beckman Coulter, California, USA).

### Cytokine gene expression following LPS stimulation

Changes in the cytokine and LPS binding receptor gene expression profile (*IL-1β, IL-2, IL-10, IFN-γ, TNF-α, TGF-β,* and *TLR-4*) in the cattle PBMCs were evaluated by quantitative reverse transcription PCR (qRT-PCR). Firstly 2 × 10^5^ PBMCs were stimulated with lipopolysaccharide (LPS; 5 µg/mL) (FUJIFILM Wako Pure Chemical, Tokyo, Japan) for 5 h and then collected for qRT-PCR. Total RNA was extracted from these stimulated PBMCs using a RNeasy Mini Kit (QIAGEN, Hilden, Germany) and cDNA was synthesized using a First Standard cDNA Synthesis Kit (Roche, Basel, Switzerland) with an anchored- oligo (dT) primer. RNA was stored at −80 °C and cDNA was stored at −30 °C until analysis. qRT-PCR was carried out using Rotor-Gene Q (QIAGEN, Hilden, Germany), and the genes were detected using a QuantiTect SYBR Green Kit (QIAGEN, Hilden, Germany). The amplification conditions were as follows: 45 cycles of 95 °C for 10 s, 60 °C for 10 s, and 72 °C for 10 s. The primer pairs used in these analyses are shown in Supplementary Table 1, and the data obtained were analyzed to the expression levels of *Glyceraldehyde 3-phosphate dehydrogenase (GAPDH*).

### Statistical analysis

Statistical analysis of colony number, rumen pH, lymphocyte subsets, and cytokine gene expression was performed by comparing data between each feeding stage of five animals with the Wilcoxon Signed-Rank Test for nonparametric analysis. Differences were considered significant when the probability value (p) was less than 5% (*p* < 0.05).

## Results

The experimental cows did not present with any clinical symptoms during the observation period. Routine blood examination revealed that all of the cows remained in the normal range of CBC results for the full course of the study.

### Intestinal microbiota

**Fig. 2:**
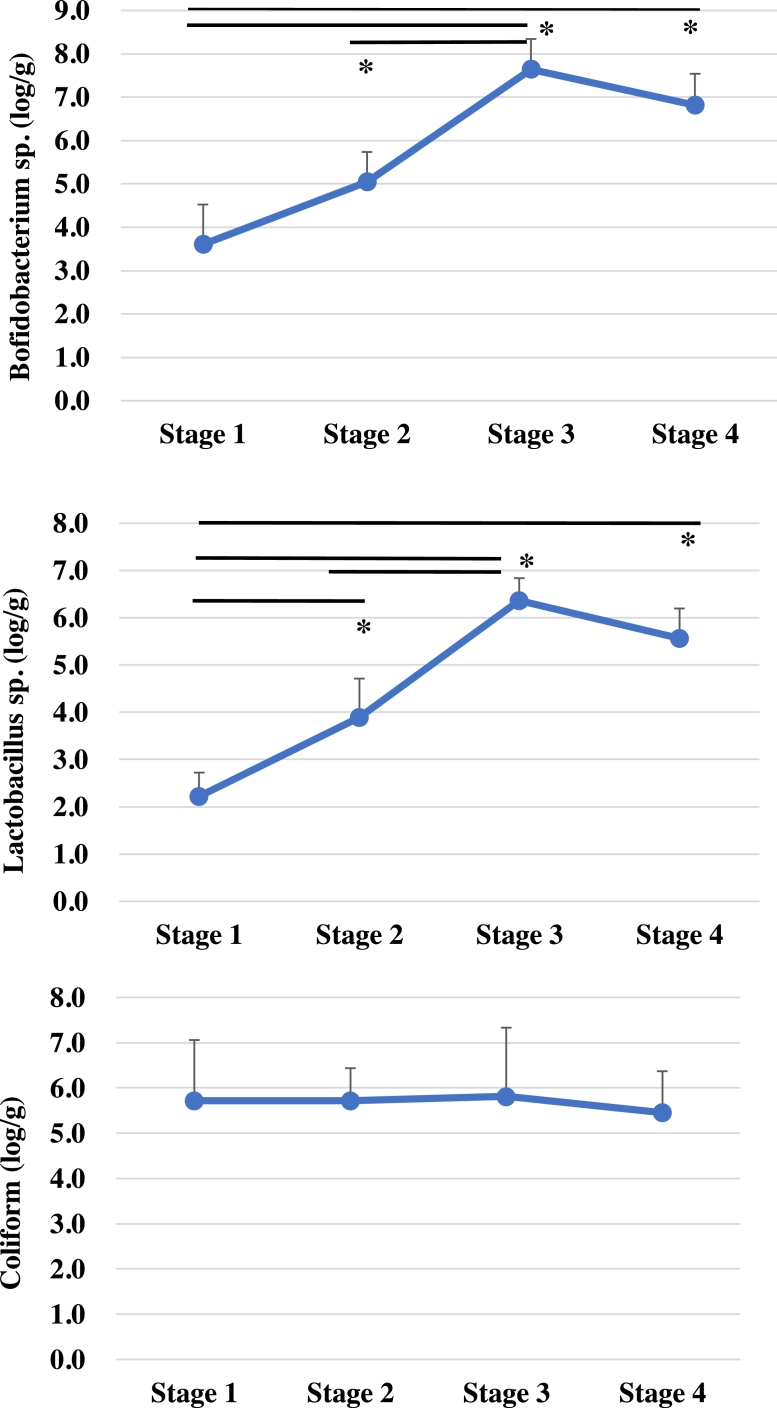
Abundance of *Bifidobacterium, Lactobacillus*, and coliforms in rectal feces samples at each stage. Changes in the number of bacteria in the feces from feed stages 1 – 4 (Lp1 administration). Significant differences between groups are indicated by * *p* < 0.05.

The relative abundance of the *Lactobacillus, Bifidobacterium*, and coliform species in each of the fecal samples from each stage are summarized in Fig. 2. The abundance of the *Bifidobacterium* (log CFU/g) at stages 1 to 4 were 3.6, 5.3, 7.6, and 6.8, respectively while the Lactobacillus were 2.2, 3.9, 6.4, and 5.6, respectively. Both the *Lactobacillus* and *Bifidobacterium* increased significantly between stages 1 and 3, but did not change significantly between stages 3 and 4. The relative abundance of the coliforms was 5.7, 5.7, 5.8, and 5.6, respectively, with these bacteria showing no response to the feeding materials or probiotic supplementation at stage 4.

### Rumen pH

**Fig. 3:**
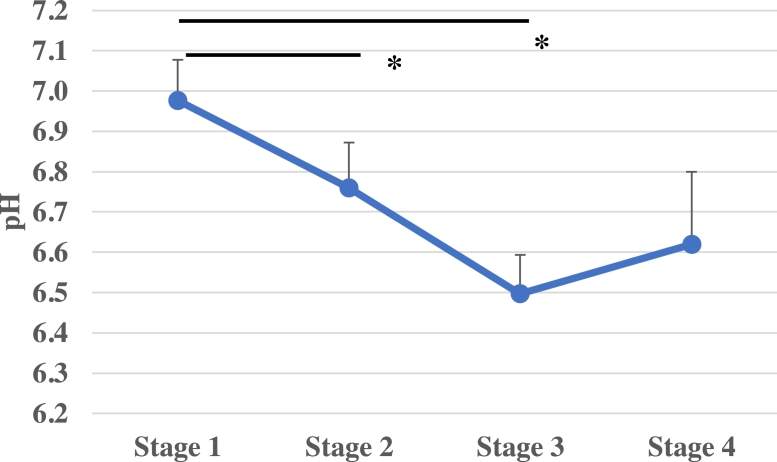
Fluctuations in lumen pH due to feed changes The average pH was measured at 3 times per day: before morning feeding, 5 h after feeding, and 10 h after feeding. The figure shows the mean and standard deviation of the five animals measured at each stage. a significant difference was confirmed between stages 2 and 3 with respect to stage 1 (* *p* < 0.05).

Rumen pH was measured at 3 points per day: after the morning feed, 5 h after feeding, and 10 h after feeding. The rumen pH (mean ± SD) at stage 1 to 4 were 7.0 ± 0.1, 6.8 ± 0.1, 6.5 ± 0.1 and 6.6 ± 0.2, respectively, thus confirming that the lumen pH decreases in response to high-energy feed (Fig. 3, *p* < 0.05).

### T lymphocyte subsets in the PBMCs

**Fig. 4:**
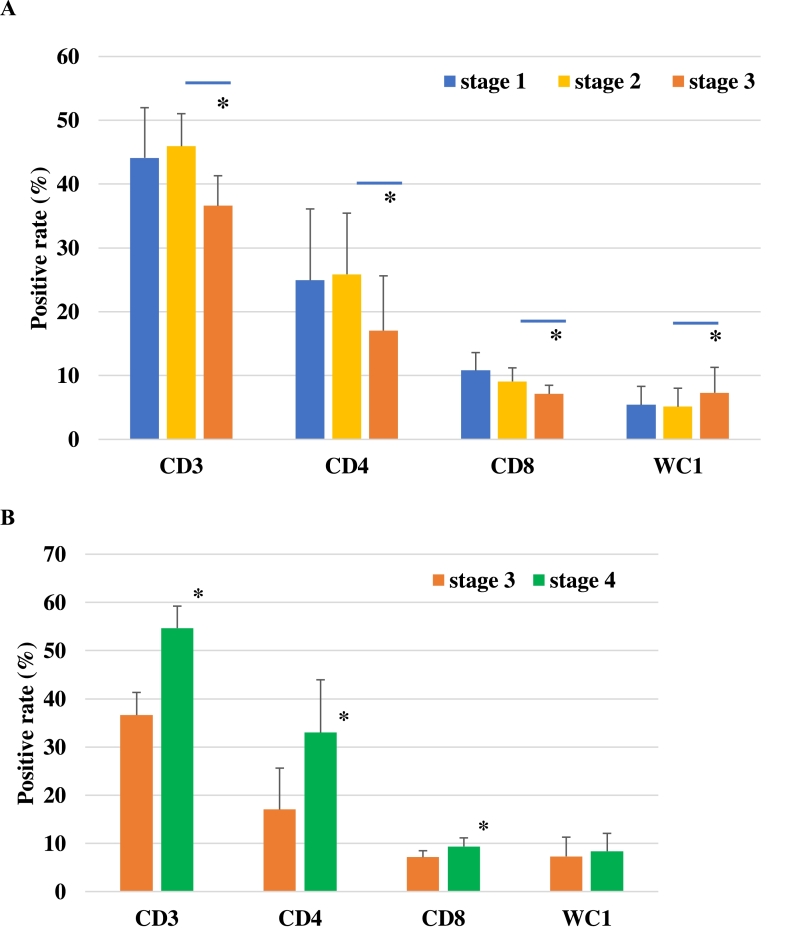
Comparison of the effects of specific dietary changes on various lymphocyte subsets. The proportion of each of the lymphocyte subsets (CD3, CD4, CD8, and WC1) in each feed group is shown. A compares the changes in each lymphocyte positive rate in each stage (1–3). B shows the change in these populations between feed stages 3 and 4 (Lp1 administration). Significant differences between groups are indicated by * *p* < 0.05.

The presence of various T lymphocyte subsets including CD3-, CD4-, and CD8 positive or WC1 positive cells were monitored by flow cytometry (Fig. 4). The proportion of CD3 positive cells at stages 1 to 3 were 44, 45, and 36% respectively while the CD4 positive rate was 25%, 26%, and 17%, respectively and the CD8 positive rates were 11%, 9%, and 7%, respectively. The number of CD3, CD4 and CD8 positive cells tended decreased between stages 1 and 3, and the significant differences were observed between stage 3 to 4 (Fig. 4A, *p* < 0.05). The proportion of WC1 positive cells in these cows at stages 1 to 3 were 5%, 5%, and 7%, respectively. A significant increase in WC1 was observed between stages 3 and 4. The cows under LP1-treated stage 4 showed a significant increase in lymphocyte subsets (CD3, CD4 and CD8) that were decreased in stage 3. However, no significant change was observed in WC1 positive ratio (Fig. 4B).

### Cytokine gene expression in LPS stimulated PBMCs

A summary of the cytokine gene (*IL-1β, IL-2, IL-10, IFN-γ, TNF-α,* and *TGF-β*) expression values in LPS stimulated PBMCs is shown in Table 2. *IL-1β, IL-2* and *IL-10* expression (mean±SD) was shown to increase significantly at stage3 when compared to stage 1, and these levels also decreased significantly at stage 4. *IFN-γ, TNF-α,* and *TGF-β* did not show any significant changes in expression between stages 1 and 4.

**Table 2:** Relative expression of various cytokines in PBMCs.

	Stage 1	Stage 2	Stage 3	Stage 4
*IFN-γ*	1.4 ± 1.1	1.1 ± 0.9	0.7 ± 0.2	0.6 ± 0.5
*IL-2*	1.8 ± 0.6	3.3 ± 1.3	8.0 ± 1.6*	4.3 ± 2.2†
*TNF-α*	0.8 ± 0.3	1.5 ± 0.2	1.2 ± 0.3	1.2 ± 0.5
*IL-1β*	1.1 ± 0.5	0.8 ± 0.4	2.6 ± 0.6*	0.9 ± 0.5†
*IL-10*	0.6 ± 0.3	0.8 ± 0.5	1.5 ± 0.6*	0.4 ± 0.2†
*TGF-β*	1.3 ± 0.5	1.8 ± 0.9	2.0 ± 0.9	1.4 ± 0.5†

⁎ *p* < 0.05, Significant differences in cytokine expression between stage 2 and 3.

† *p* < 0.05, Significant differences in cytokine expression between stage 3 and 4.

Supplementary experiment results were shown for the LPS-stimulated *IL1-β* response at 3 weeks after the Lp1 administration. The expression of *IL1-β* by LPS stimulation in PBMC derived from 5 cows increases *IL1-β* expression 3 weeks after the Lp1 treatment (Supplementary figure 1). Therefore, the suppression of *IL1-β* expression was shown to be released in PBMC after the end of the LP1 treatment period, indicating that the LP1 effect was transient for the treatment period.

## Discussion

Dairy cows have high nutritional needs, but the high energy feeding compromises rumen health, causing subacute ruminal acidosis (SARA), following with physical failure (Humer, et al. 2018). In high producing dairy herds, proper feeding management is required to maintain rumen health and prevent disease. Previous studies have suggested that supplementation with feed additives containing probiotics may reduce disease incidence in cow (Vibhute, et al., 2011). In this study, we monitored changes in immune function in cows during transitional feeding and examined the effects of functional *Lactobassillus* Lp1 administration during high-energy feeding period. To examine the immunological changes associated with the transition from low to high-energy diets (stages 1–3), five cows were fed each stage of the diet, and the individual changes were examined. In addition, Lp1 was administered to the cows under a high-energy diet (stage 4), and changes in the rumen pH of the dairy cows were observed, as well as changes in the intestinal microflora and the immune response at the cellular level.

The transition of *lactobacillus* sp. in feces showed a clear change with transitional feeding; *Lactobacillus sp*. and *Bifidobacterium sp*. increased significantly from stage 1 to 3. This clearly indicated that the diet composition was favorable for the growth of lactic acid bacteria. Coliform, on the other hand, did not change significantly with transitional feeding. It may be part of the bacterial flora that is not affected by transitional feeding in these experiments.

In general, rumen pH is maintained by the secretion of saliva in response to hay chewing and rumination, allowing for the maintenance of the commensal bacteria balance in the microbiome (Mackie & Gilchrist, 1979). Changes in the feed composition affected the numbers of *Lactobacillus* and *Bifidobacterium* spp. In experimental cows, increased feed TDN levels (decreased Hay consumption and increased grain feed rates) resulted in a significant increase in the number of *Lactobacillus* and *Bifidobacterium* spp. in the gastrointestinal tract. On the other hand, the pH in the rumen was significantly decreased in response to increasing TDN levels, which is in agreement with a previous report (Neubauer et al., 2018). Here, we observed that rumen pH decreased in response to high energy feeding (stage 3). A comparison of rumen pH trends from stage 1–3 showed a significant decrease to pH of 6.5. The risk of SARA increases after a lumen pH of less than 5.6 and more than 3 h a day. (Plaizier et al. 2008). The stage 3 feeding is high energy diets, can be considered as high-risk management, however, a small increase in pH was observed in Stage 4 after administration of Lp1, indicating a possibility SARA control effect on the rumen environment. Blends of lactic acid-producing bacteria have been investigated as probiotics to be fed to high-producing dairy cows to increase pH, VFA production, and lactic acid utilization in the rumen (Neubauer et al., 2018Philippeau et al., 2017). These effects not only improve the gut microbiota, but also affect immune function.

The analysis of the lymphocyte subsets showed that CD3, CD4, and CD8 positive αβ-type T lymphocytes decrease following the addition of high TDN feeding. Lymphocyte composition is a useful indicator of immune status. We compared peripheral blood lymphocyte subsets in different feeding stages to understand the impact of the feed composition on overall immune activation. In non-parturition and non-lactating cattle, excessive energy feeding was shown to reduce αβ-type T lymphocytes. High-energy diets have been shown to lower rumen pH and alter the composition of the intestinal microbiota. In addition, high energy diets have also been linked to increases in the amount of circulating LPS and LPS induced reductions in αβ T lymphocytes (Klaudia & Alina, 2015; Ohtaki et al., 2020). Many researchers believe that high-energy feed should be considered as a background factor when evaluating immune function in cattle. In contrast, the breeding cycle has been linked to increases in the αβ T lymphocytes in the postpartum and early lactation phases (Meglia, Johannisson, Agenäs, Holtenius & Waller, 2005). Peripheral lymphocytes are known to exhibit complex responses to the ecological stresses induced during pregnancy, labor, and lactation. Dairy cows are moved onto a high energy diet following the establishment of a milking program after calving, with many of these animals developing a variety of metabolic and inflammatory diseases, such as fatty liver disease, during this period (Katoh, 2002). The results of this study suggest that the switch to a high-energy feed tends to decrease the lymphocyte ratios and enhances the inflammatory response to LPS stimulation. From the perspective of liver disease such as non-alcoholic steatohepatitis, the inflammatory cytokines (*IL-1β, IL-2,* and *TNF-α*) are produced in the liver and are associated with disease (Bruscalupi, Agostinelli, Stronati & Cucchiara, 2015). Inflammation of the liver is one of the causes of liver disease as well as ruminants. Thus, revealing that these lactating cows manifest several background factors that increase their risk for developing several immune-mediated disorders.

The increase in CD3, CD4, and CD8 positive cells during high energy feeding in response to LP1 supplementation and the return of the cytokine expression levels to that of low energy diet suggests that LP1 supplementation may protect the unbalanced immune response associated with high energy diets. In the LPS stimulation study using PBMC from five cows, an increase in inflammatory cytokine expression was observed following with increasing feed energy, but this was attenuated by LP1 administration. Suppressive effect of inflammation was observed with LP1 administration, although it was only a part of the systemic response since only PBMC was used. A previous study in human NK cells showed that supplementation with *Lactobacillus plantarum* promoted *IL-10* production (Qiu et al., 2017). However, the increase in anti-inflammatory cytokines (*IL10 and TGFβ*) was not observed with LP1 administration, which may indicate that the inflammatory regulatory response was already affecting PBMC in the LP1-treated cows. Alternatively, it could be due to other than inflammatory cytokine regulatory mechanisms. One of the possible explanations is that butyrate production, which is associated with the improvement of intestinal microflora by LP1 administration, reduced inflammation. In addition, the suppression of *IL-1β* expression by LPS stimulation disappeared after LP1 administration was terminated, suggesting that LP1-producing molecules may act as transcription factor regulators (supplementary figure 1). Short-chain fatty acids produced by enteric bacteria, including Lactobacillus, act on cells by means of G-protein-coupled receptors (GPCRs) on the cell surface. Butyrate promotes the induction of T cell differentiation into regulatory T cells via GPR43 (Furusawa et al., 2013). In addition, it regulates *NF-κB* activation via TLR signaling such as lipopolysaccharide (Kellow, Coughlan & Reid, 2014; Vinolo, Rodrigues, Nachbar & Curi, 2011). Since there are many unanswered questions about the LP1-producing molecules and their mechanisms, further analysis is needed.

In conclusion, A dietary energy transfer feeding study was conducted in cattle. The dietary composition changes associated with this continuous energy shift were shown to have immune consequences in cattle. In particular, the high-energy diet decreased the percentage of αβ T lymphocytes and enhanced *IL-1β* and *IL-2* expression in LPS-stimulated PBMC, indicating a risk of increased inflammation. The functional *Lactobacillus plantarum* LP1 administration is expected to have an immunomodulatory function in alleviating excessive LPS-stimulated inflammatory responses and decreasing lymphocyte levels in cows under high-energy diets. The application of functional probiotics in the feeding management of high producing cows may be a tool to be considered as an option to mitigate the associated risk of deterioration in cow metabolism and immune function.

## Appendix Supplementary materials

Image, image 1

Image, application 2

## References

[bib0001] Andersson L. (1988). Subclinical ketosis in dairy cows. The Veterinary Clinics of North America. Food Animal Practice.

[bib0002] Bruscalupi G., Agostinelli L., Stronati L., Cucchiara S. (2015). LPS-induced TNF-α factor mediates pro-inflammatory and pro-fibrogenic pattern in non-alcoholic fatty liver disease. Oncotarget.

[bib0003] Casper D.P., Hultquist K.M., Acharya I.P. (2021). Lactobacillus plantarum GB LP-1 as a direct-fed microbial for neonatal calves. Journal of Dairy Science.

[bib0004] Colombo E.A., Cooke R.F., Brandão A.P., Wiegand J.B., Schubach K.M., Sowers C.A., G.ouvêa V.N. (2021). Performance, health, and physiological responses of newly received feedlot cattle supplemented with pre- and probiotic ingredients. Animal : An International Journal of Animal Bioscience.

[bib0005] Fuller R. (1989). Probiotics in man and animals. The Journal of Applied Bacteriology.

[bib0006] Furusawa Y., Obata Y., Fukuda S., Endo T.A., Nakato G., Takahashi D. (2013). Commensal microbe-derived butyrate induces the differentiation of colonic regulatory T cells. Nature.

[bib0007] Harder I., Stamer E., Junge W., Thaller G. (2019). Lactation curves and model evaluation for feed intake and energy balance in dairy cows. Journal of Dairy Science.

[bib0008] Hua X., Goedert J.J., Pu A., Yu G., Shi J. (2016). Allergy associations with the adult fecal microbiota: Analysis of the American gut project. EBioMedicine.

[bib24] Humer E (2018). Signals for identifying cows at risk of subacute ruminal acidosis in dairy veterinary practice. Journal of animal physiology and animal nutrition. Journal of dairy science.

[bib0009] Katoh N. (2002). Relevance of apolipoproteins in the development of fatty liver and fatty liver-related peripartum diseases in dairy cows. The Journal of Veterinary Medical Science / The Japanese Society of Veterinary Science.

[bib0010] Kayasaki F., Okagawa T., Konnai S., Kohara J., Sajiki Y., Watari K. (2021). Direct evidence of the preventive effect of milk replacer-based probiotic feeding in calves against severe diarrhea. Veterinary Microbiology.

[bib0011] Kellow N.J., Coughlan M.T., Reid C.M. (2014). Metabolic benefits of dietary prebiotics in human subjects: A systematic review of randomised controlled trials. British Journal of Nutrition.

[bib0012] Klaudia C., Alina W. (2015). The influence of enrofloxacin, florfenicol, ceftiofur and E. coli LPS interaction on T and B cells subset in chicks. Veterinary Research Communications.

[bib0013] Krizsan S.J., Sairanen A., Höjer A., Huhtanen P. (2014). Evaluation of different feed intake models for dairy cows. Journal of Dairy Science.

[bib0014] Mackie R.I., Gilchrist F.M.C. (1979). Changes in lactate-producing and lactate-utilizing bacteria in relation to pH in the rumen of sheep during stepwise adaptation to a high-concentrate diet. Applied and Environmental Microbiology.

[bib0015] Meglia G.E., Johannisson A., Agenäs S., Holtenius K., Waller K.P. (2005). Effects of feeding intensity during the dry period on leukocyte and lymphocyte sub-populations, neutrophil function and health in periparturient dairy cows. Veterinary Journal (London, England : 1997).

[bib0016] Mitsuoka T. (2014). Establishment of intestinal bacteriology. Bioscience of Microbiota, Food and Health.

[bib0017] Neubauer V., Petri R., Humer E., Kröger I., Mann E., Reisinger N. (2018). High-grain diets supplemented with phytogenic compounds or autolyzed yeast modulate ruminal bacterial community and fermentation in dry cows. Journal of dairy Science.

[bib0018] Ohtaki T., Ogata K., Kajikawa H., Sumiyoshi T., Asano S., Tsumagari S. (2020). Effect of high-concentrate corn grain diet-induced elevated ruminal lipopolysaccharide levels on dairy cow liver function. The Journal of Veterinary Medical Science / The Japanese Society of Veterinary Science.

[bib26] Philippeau C (2017). Effects of bacterial direct-fed microbials on ruminal characteristics, methane emission, and milk fatty acid composition in cows fed high-or low-starch diets. Journal of dairy science.

[bib0019] Qiu Y., Jiang Z., Hu S., Wang L., Ma X., Yang X. (2017). Lactobacillus plantarum enhanced IL-22 production in Natural Killer (NK) cells that protect the integrity of intestinal epithelial cell barrier damaged by enterotoxigenic escherichia coli. International Journal of Molecular Science.

[bib0020] Ringseis R., Gessner D.K., Eder K. (2015). Molecular insights into the mechanisms of liver-associated diseases in early-lactating dairy cows: Hypothetical role of endoplasmic reticulum stress. Journal of Animal Physiology and Animal Nutrition (Berl)..

[bib25] Vibhute V (2021). Effect of probiotics supplementation on the performance of lactating crossbred cows. Veterinary World.

[bib0021] Vinolo M.A., Rodrigues H.G., Nachbar R.T., Curi R. (2011). Regulation of inflammation by short chain fatty acids. Nutrients.

[bib0022] Weiss W.P. (2017). A 100-year review: From ascorbic acid to zinc—Mineral and vitamin nutrition of dairy cows. Journal of dairy science.

[bib0023] Welch C.B., Lourenco J.M., Davis D.B., Krause T.R., Carmichael M.N., Rothrock M.J. (2020). The impact of feed efficiency selection on the ruminal, cecal, and fecal microbiomes of angus steers from a commercial feedlot. Journal of animal science.

