# Supplementary figures and images for: Changes in immune system and intestinal bacteria of cows during the transition period
Source: Vet Anim Sci. 2021 Dec 2;14:100222. doi: 10.1016/j.vas.2021.100222 (PMC8666551; doi:10.1016/j.vas.2021.100222)

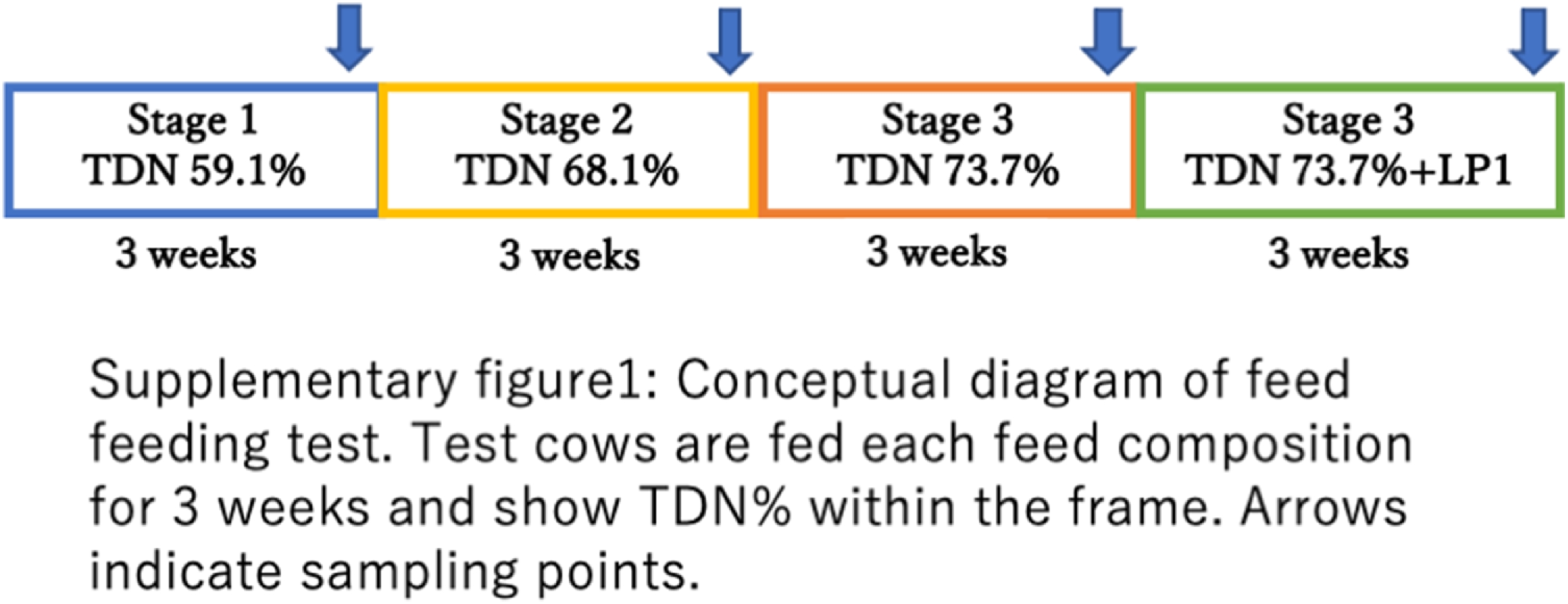

Supplement: Supplementary file 1 [file mmc1.jpg]
