# Supplementary material for: Changes in immune system and intestinal bacteria of cows during the transition period
Source: Vet Anim Sci. 2021 Dec 2;14:100222. doi: 10.1016/j.vas.2021.100222 (PMC8666551; doi:10.1016/j.vas.2021.100222)
Supplement: Supplementary file 2 [file mmc2.docx]

Supplementary Table 1. Description of all the primers used in the qRT-PCR analysis

| Target gene | Sequences (5’ -3’) | Tm |
| --- | --- | --- |
| *GAPDH* | Forward: 5’-GATTGTCAGCAATGCCTCCT-3’ | 59.9 |
|  | Reverse: 5’-GGTCATAAGTCCCTCCACGA-3’ | 60.3 |
| *IL-1β* | Forward: 5’-TTCTCTCCAGCCAACCTTCATT-3’ | 61.4 |
|  | Reverse: 5’-ATCTGCAGCTGGATGTTTCCAT-3’ | 62.1 |
| *IL-2* | Forward: 5’-TGCACTAACTCTTGCACTCG-3’ | 59.6 |
|  | Reverse: 5’-ACCTTGGGCACGTAAAAGTC-3’ | 60.0 |
| *IL-10* | Forward: 5’-TGACATCAAGGAGCACGTGAA-3’ | 61.5 |
|  | Reverse: 5’-TCTCCACCGCCTTGCTCTT-3’ | 63.3 |
| *IFN-γ* | Forward: 5’-AGGTCATTCAAAGGAGCATGGA-3’ | 61.5 |
|  | Reverse: 5’-TTATGGCTTTGCGCTGGA-3’ | 59.2 |
| *TNF-α* | Forward: 5’-CAAGTAACAAGCCGGTAGC-3’C | 60.3 |
|  | Reverse: 5’-TGGAAGACTCCTCCCTGGTA-3’ | 61.2 |
| *TGF-β* | Forward: 5’-TTCTTCAACACGTCCGAGCTC-3’ | 61.9 |
|  | Reverse: 5’-AGCGCCAGGAATTGTTGCTAT-3’ | 62.4 |
| *TLR−４* | Forward: 5’-ACTGACGGGAAACCCTATCC-3’ | 57.5 |
|  | Reverse: 5’-CAGGTTGGGAAGGTCAGAAA-3’ | 55.4 |
